# Human behavior recognition based on sparse transformer with channel attention mechanism

**DOI:** 10.3389/fphys.2023.1239453

**Published:** 2023-11-02

**Authors:** Keyan Cao, Mingrui Wang

**Affiliations:** School of Computer Science and Engineering, Shenyang Jianzhu University, Shenyang, Liaoning, China

**Keywords:** human activity recognition, wearable biosensors, sparse transformer, attention, time series

## Abstract

Human activity recognition (HAR) has recently become a popular research field in the wearable sensor technology scene. By analyzing the human behavior data, some disease risks or potential health issues can be detected, and patients’ rehabilitation progress can be evaluated. With the excellent performance of Transformer in natural language processing and visual tasks, researchers have begun to focus on its application in time series. The Transformer model models long-term dependencies between sequences through self-attention mechanisms, capturing contextual information over extended periods. In this paper, we propose a hybrid model based on the channel attention mechanism and Transformer model to improve the feature representation ability of sensor-based HAR tasks. Extensive experiments were conducted on three public HAR datasets, and the results show that our network achieved accuracies of 98.10%, 97.21%, and 98.82% on the HARTH, PAMAP2, and UCI-HAR datasets, respectively, The overall performance is at the level of the most advanced methods.

## 1 Introduction

With the continuous advancement of artificial intelligence and wearable technology, research on human activity recognition (HAR) based on inertial measurement unit (IMU) devices has become a trending field (Yadav et al., 2021). It has extensive practical applications such as smart homes, health monitoring, exercise tracking, and game design (Rashidi and Cook, 2009; Hong et al., 2010; Ke et al., 2013). Compared to visual-based HAR, sensor-based HAR methods can better protect user privacy, especially with the development and proliferation of wearable devices that embed sensors such as accelerometers, gyroscopes, and magnetometers.

Deep learning has been widely applied in the HAR field in recent years (Zhang et al., 2022). Deep learning models do not rely on prior knowledge and avoid manual feature extraction, which can automatically learn more advanced or meaningful features. Convolutional neural networks (CNNs) have completed various HAR tasks (Jiang and Yin, 2015; Teng et al., 2020; K. Wang, He, and Zhang, 2019; Zeng et al., 2014). CNN models can automatically extract multi-level feature representations from sensor signals (Jiang and Yin, 2015), improving human activity recognition accuracy. However, CNNs usually need to convert input data into image form, which is not the optimal representation for some signal data types, such as time series or waveform signals. They may cause information loss or noise increase. Standard deep neural networks, including CNNs and recurrent neural networks (RNNs), have limitations in HAR tasks (Gao et al., 2021). As the number of sensors increases, the size of the convolutional layer may ignore features in specific dimensions, affecting the algorithm’s accuracy (Szegedy et al., 2015).

The Transformer model (Vaswani et al., 2017) proposal combines the advantages of CNNs and RNNs. The Transformer model can learn more extended sequence contextual information and has better parallel performance in computation. Transformer models have been widely used in the image domain (Dosovitskiy et al., 2021) and natural language processing (Devlin et al., 2019). The Transformer model captures long-term dependencies between sequences through self-attention mechanisms, allowing it to capture contextual information over extended periods (Dai et al., 2019). The signals collected by sensors are based on time-series data, and the self-attention mechanism in the Transformer model can consider information from all positions in the sequence simultaneously, enabling parallel computation and improving efficiency (Reza et al., 2022). Compared with complex CNNs and RNNs models, Transformer models are more stable (Vaswani et al., 2017).

However, existing deep learning methods such as CNNs, RNNs, and Transformers still have some limitations. Firstly, most existing HAR classification models have many parameters for training, leading to poor performance in natural environments. Secondly, existing models need to consider the different importance levels of data on different channels of sensor-collected data. Moreover, existing Transformer-based algorithms have many parameters, placing higher performance requirements on mobile and wearable devices with low power consumption.

This paper proposes a lightweight Transformer model incorporating channel attention mechanisms to address these issues.

1. For HAR scenarios, we propose an input structure based on channel attention mechanisms for 1D CNNs, enhancing the model’s ability to extract features from data on different channels.
2. Instead of the traditional Transformer model’s self-attention mechanism. We propose HAR-Attention mechanism reduces the FLOPs calculation of the model by using a sparse self-attention matrix composed of Slide Attention and Random Attention while ensuring the model’s local sensitivity and global feature extraction capabilities.
3. We conducted extensive experiments on three public datasets, demonstrating the robustness and practicality of the proposed mode.

The rest of this paper is organized as follows. Section 2 reviews the development of HAR algorithm models, Section 3 introduces the details of the proposed model, Section 4 describes the experimental setup, and results in detail.

## 2 Related works

This section reviews the current work related to HAR. Human activity recognition (HAR) on mobile devices based on inertial measurement units (IMUs) is the task of identifying daily human activities (such as walking, running, and jumping) based on time-series data readings. In this field, a combination of accelerometers and gyroscopes is commonly used, where the accelerometer is a sensor that provides a detailed description of acceleration, and the gyroscope is a device that senses angular velocity.

### 2.1 HAR based on traditional deep learning

Research on deep learning-based feature extraction has received wide attention in recent years. CNN and RNN are usually superior in feature extraction ability to traditional feature selection and machine learning techniques. CNN is very suitable for handling time-series data used in HAR tasks (Gu et al., 2021). Gao et al. (2021) found that compared with CNN, the feature representation ability of RNN is weaker, and existing HAR attention mechanisms mainly focus on temporal continuity and cannot balance the spatiotemporal dependencies of multimodal sensing signals well.

The CNN model achieved significant results in HAR on sensors and outperformed other state-of-the-art algorithms requiring advanced preprocessing or tedious manual feature extraction. For example, Zeng et al. (2014) first applied CNN to HAR and extracted the acceleration time series’ local dependencies and scale-invariant characteristics. Jiang and Yin (2015) converted raw sensor signals into 2D image signals and then classified the signal images using two layers of CNN to achieve the desired activity recognition. Teng et al. (2020) combined local loss and hierarchical training mechanisms into a deep HAR classifier. They proposed a hierarchical CNN that uses a local loss function, improving the memory efficiency of HAR applications. K. Wang, He, and Zhang (2019) proposed an attention-based CNN to perform weakly labeled HAR tasks, promoting the annotation process of sensor data. The LSTM model (Hua et al., 2019) can learn the time correlation between data. Bokhari et al. (2021) used a simplified network-gated recurrent unit (GRU) of LSTM with fewer parameters and faster convergence speed. However, the recognition effect is similar to that of LSTM. Yin et al. (2022) used a hybrid of multiscale CNN and Bi-LSTM to improve feature extraction and performance. Ordóñez and Roggen (2016) proposed a general deep framework for activity recognition composed of CNN and long short-term memory (LSTM), which can further fuse multimodal sensors to improve the classification accuracy of CNN.

### 2.2 HAR based on attention mechanism

CNN has become the dominant technology in computer vision, and many researchers continue to study it to improve its performance. The introduction of attention mechanisms into convolutional modules has shown great potential in improving the performance of models, attracting the attention of many researchers (Niu, Zhong, and Yu, 2021). Squeeze-and-Excitation Networks (SENet) (Hu, Shen, and Sun, 2018) is one of the usual methods, which brings significant performance gains to various CNN architectures by incorporating channel attention into convolution blocks. The Transformer architecture is a deep neural network initially developed for natural language processing. They were designed to address the problems faced by RNN architectures and use self-attention mechanisms and similarity feature scores (Rumelhart, Hinton, and Williams, 1986), which are the core innovations of the Transformer. The Transformer model shows that attention mechanisms do not require recursive units to achieve equivalent performance. In addition, Transformer models are more easily parallelizable than CNN models. Mahmud et al. (2020) combined sensor-modal attention, self-attention, and global temporal attention to generating high-dimensional feature representations for classification. Khan and Ahmad (2021) used a multi-head convolutional neural network with added SENet attention to solving the wearable activity recognition problem. Tang et al. (2022) captured the interrelationships between the three dimensions of sensors, time, and channels by establishing triple attention. SHAVIT and KLEIN (Shavit and Klein, 2021) first introduced the Transformer model into human behavior recognition and proposed methods that showed better prediction accuracy than CNN-based solutions and could be better transferred between datasets. Iveta and Martin (Dirgová Luptáková, Kubovčík, and Pospíchal, 2022) used Transformers to obtain time correlations of feature sequences and added attention mechanisms to highlight intrinsic features.

Based on reading and summarizing a large number of literature studies in the field of human behavior recognition, we proposes a hybrid model based on channel attention mechanism and sparse attention mechanism transformer. The roles of channel attentional mechanisms and sparse attentional mechanism transformer are also discussed in the context of ablation experiments. The model is used for the task of human activity recognition from wearable device sensor data and effectively improves the accuracy of sensor-based HAR.

## 3 Proposed framework

**FIGURE 1:**
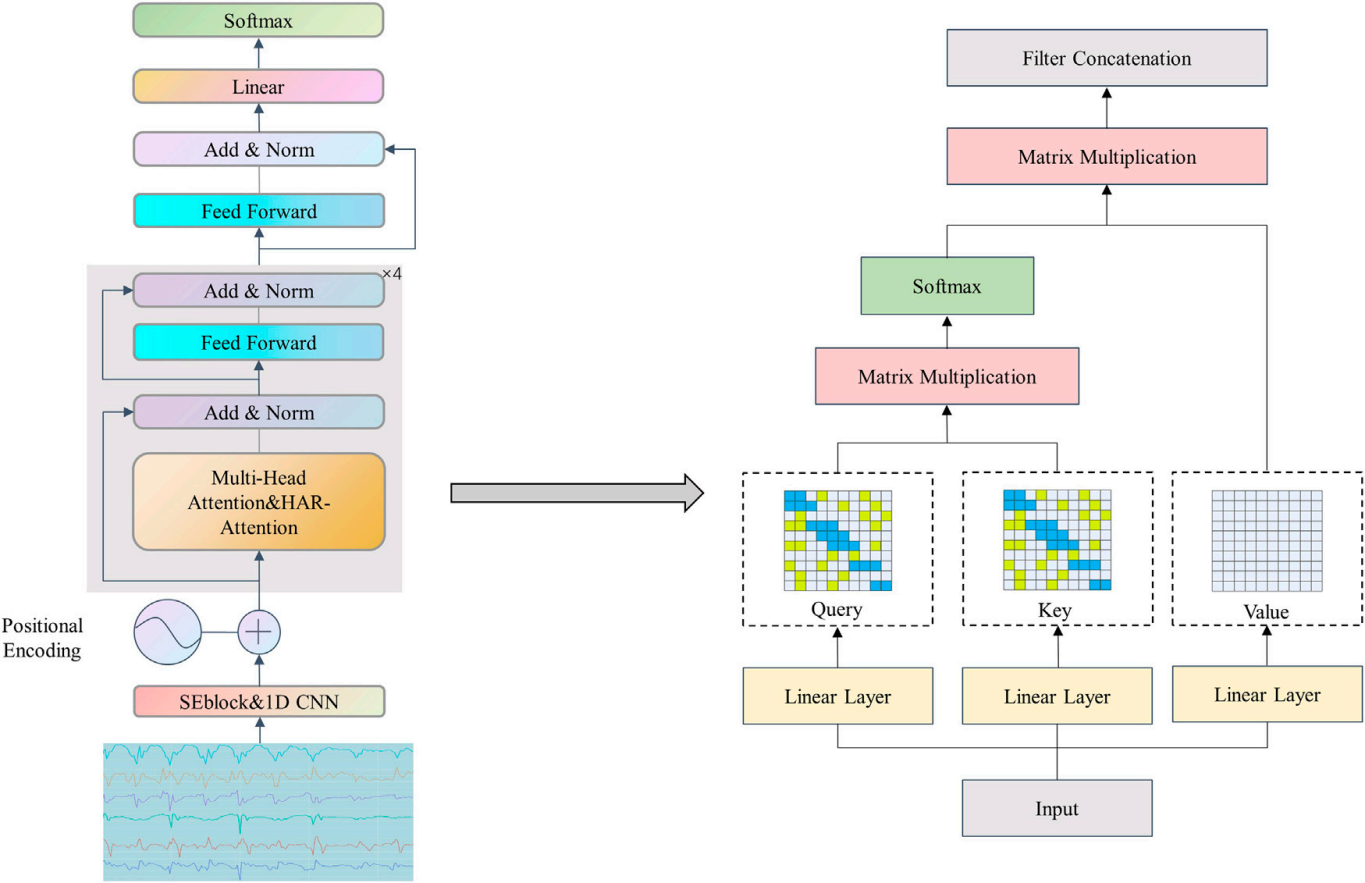
The overview of our network.

In this section, we aim to propose a hybrid model based on a channel attention mechanism for 1D CNN and Transformer, to improve the accuracy of human activity recognition based on inertial sensors as much as possible while reducing the Flops computation of the Transformer through sparse attention mechanism. Some models in existing methods exhibit suboptimal levels of accuracy, while certain other models fail to address the issue of non-parallel processing for multi-sensor data. Furthermore, the redundancy resulting from self-attention mechanisms has not been adequately considered when deploying Transformer-based models. To achieve this goal, this paper proposes the following model, as shown in Figure 1, which uses multi-dimensional signal data collected by inertial sensors to automatically recognize the wearer’s movements.

The proposed processing framework includes several components: first, noise removal and motion window segmentation are performed, and then initial feature extraction is performed through channel attention of 1D CNN based on the channel attention mechanism, which can better capture differences and correlations between different channels. The extracted features are input to the backbone network Transformer for further context feature extraction. The Flops computation of the Transformer model is reduced through a sparse attention mechanism as shown in the right of Figure 1. Finally, classification is performed through fully connected layers. The specific steps of each module will be discussed in the following sections.

### 3.1 Data segmentation

After preprocessing the data, it must be segmented before inputting into the model. The sliding window technique is widely used for sensor-based HAR in the data segmentation process. The length and overlap rate of the sliding window has some influence on the recognition accuracy. However, there has yet to be a consensus on the sliding window’s optimal length and overlap rate. Long sliding windows contain more sensor data, resulting in higher recognition accuracy and more conducive to identifying some complex activities, but the recognition speed will decrease. Smaller windows are suitable for recognizing faster activities. Most literature sets the two sliding window parameters consistent with previous research. In this paper, for fairness, we also set the length and overlap rate of the sliding window to be consistent with other literature.

### 3.2 Model details

The model proposed in this paper is based on the Transformer architecture (Vaswani et al., 2017), a well-known sequence-to-sequence model in the field of NLP, consisting of an encoder and decoder part. Similarly, the model can be applied to the field of sensor-based HAR. Different from the direct use of the Transformer model in the field of human behavior recognition by Dirgová Luptáková, Kubovčík, and Pospíchal (2022). We modified the input embedding and self-attention mechanism to use Transformer efficiently in our application. First, we proposed an input structure based on channel attention mechanism 1D CNN to enhance further the feature extraction ability of HAR signals instead of the 1D CNN in the model (Shavit and Klein, 2021). Secondly, to solve the problem of large-scale Flops, we proposed a more precise and powerful attention mechanism structure to replace the self-attention part in the original model; namely, we embedded two-level attention mechanisms in the model: local attention mechanism in Slide Attention and global attention mechanism in Random Attention. For a given sequence X = (x1, x2, … , xt), where t represents the length of the given sequence, the Transformer outputs a vector P = (p1, p2, p3 … , pi), where pi represents the probability that the sequence is classified as class i. Figure 1 describes the architecture of the proposed model.

#### 3.2.1 Fusion of channel attention mechanism into 1D CNN

In the Transformer model, word embedding is an essential step at the beginning of the model. To map each point at each position to a number, a structure is proposed to replace the word embedding in the Transformer model, which uses 1D CNN convolutional layers, and the size of the input and output of this layer are the same (Shavit and Klein, 2021). However, using only multiple convolutional layers limits its ability to extract motion features. In the multi-dimensional acceleration signal collected by inertial sensors, the importance of each channel is different, and extracting key channels and performing feature extraction can enhance the model’s learning ability for signal features.

**FIGURE 2:**
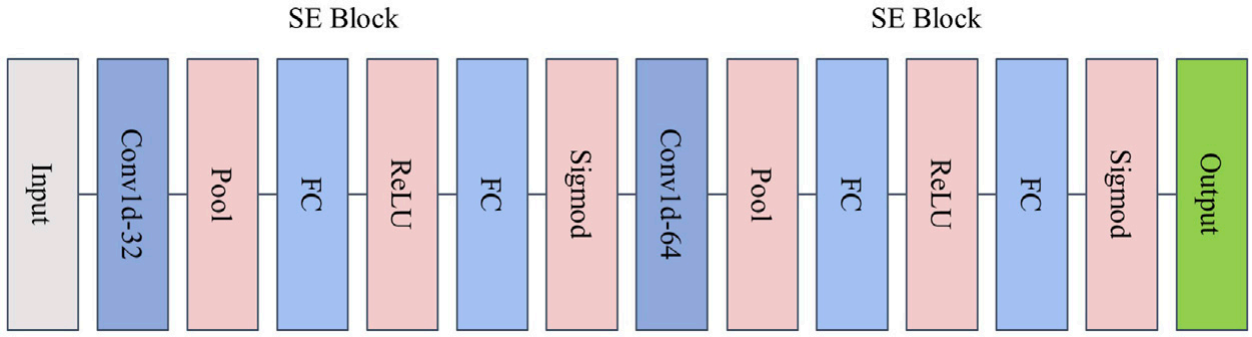
SE-Block and CNN.

To solve this problem, we proposed an input structure based on channel attention mechanism 1D CNN to enhance the ability to extract motion features from acceleration signals collected by inertial sensors. In the proposed model, the channel attention mechanism is embedded into the 1D CNN model, which selects channels with more vital representation ability from multiple channels in the input and combines them with the 1D CNN network to extract local morphological features. Figure 2 shows the proposed attention architecture.

First, for the input to the model is the three-axis acceleration data collected in the pre-processed inertial sensors, given a data sample $X\in R^{1\times n\times t}$ (where n is the data length and t is the number of channels). The input channel feature space $X=\left(x1,x2,...,\text{xt}\right)$ is then subjected to a global level pooling operation to obtain the compressed feature information, where t denotes the number of channels and the size of each channel is 1×n .

$$S\left(X\right)=\frac{1}{n}\sum_{i=1}^{n}\ln\left(i\right)$$

where n denotes the length of the feature vector. Subsequently, the compressed feature information is used to perform the excitation operation. The channel weights are generated, and the obtained weights are adaptively calibrated by two nonlinear fully connected (FC) layers and the Sigmoid function. The learned weights are multiplied onto the corresponding channel feature vector X to obtain the weighted information channels.

#### 3.2.2 Position encoding

To effectively process inertial sensor signals, the proposed model in this paper needs to encode position information and embed it into the feature vectors obtained from the previous step. As the waveform of HAR signals is periodic, even the same value has different meanings at different positions over some time. We first use a lookup table for position encoding, and the calculation process can be completed in $O\left(1\right)$ . This processing time allows longer sequence lengths to be processed during training. The specific formula is as follows:

$$\begin{array}{c} PE_{\left(pos,2i\right)}=\sin\left({\left(\frac{pos}{10000}\right)}^{\frac{2i}{d_{model}}}\right)\\ PE_{\left(\text{pos},2i+1\right)}=\cos\;\left({\left(\frac{pos}{10000}\right)}^{\frac{2i}{d_{model}}}\right) \end{array}$$

Where pos is the position, and i is the dimension size. According to the above formula, each dimension of position encoding corresponds to a sine curve. The wavelength forms a geometric series from 2π to 10000*2π . We chose this function because it allows the model to learn features based on relative position.

#### 3.2.3 Transformer encoder

In human behavior recognition, context information of temporal sequences can reveal rich features. Therefore, the Transformer model, which is good at capturing context information, is used as the backbone network of the proposed model in this paper.

In the Transformer model, the self-attention mechanism is an important part. When calculating multi-head self-attention, the window information is first converted into three vectors $Q_{h}$ , $K_{h}$ and $V_{h}$ through linear transformations shown in formula (),

$$\begin{array}{c} Q_{h}=QW_{h}^{Q}\in R^{t\times d^{\prime }}\\ K_{h}=KW_{h}^{K}\in R^{t\times d^{\prime }}\\ V_{h}=VW_{h}^{V}\in R^{t\times d^{\prime }} \end{array}$$

where d represents the dimension of the vectors, $d^{\prime }=\frac{d}{n_{h}}$ and $n_{h}$ is the number of heads. Matrices $W_{h}^{Q},$ $W_{h}^{K}$ , $W_{h}^{V}\in R^{t\times d^{\prime }}$ are linear projections from d to $d^{\prime }$ . After obtaining the three linear transformations, the self-attention relationship matrix is calculated using the following formula (),

$$h\left(Q,K,V\right)=\text{softmax}\left(\frac{Q_{h}\left(K_{h}^{T}\right)}{\sqrt{d}}\right)V_{h}\in R^{t\times d^{\prime }}$$

where the network output is $h\left(Q,K,V\right)$ , a weighted sum of $Q_{h}$ , $K_{h}$ and $V_{h}$ . The dot product of $Q_{h}$ and $K_{h}$ is calculated, and then the obtained parameters are normalized by the softmax function and multiplied by these values $V_{h}$ . The softmax function is a classifier used for prediction, and its output value determines the probability of different classes.

### 3.3 HAR attention

**FIGURE 3:**
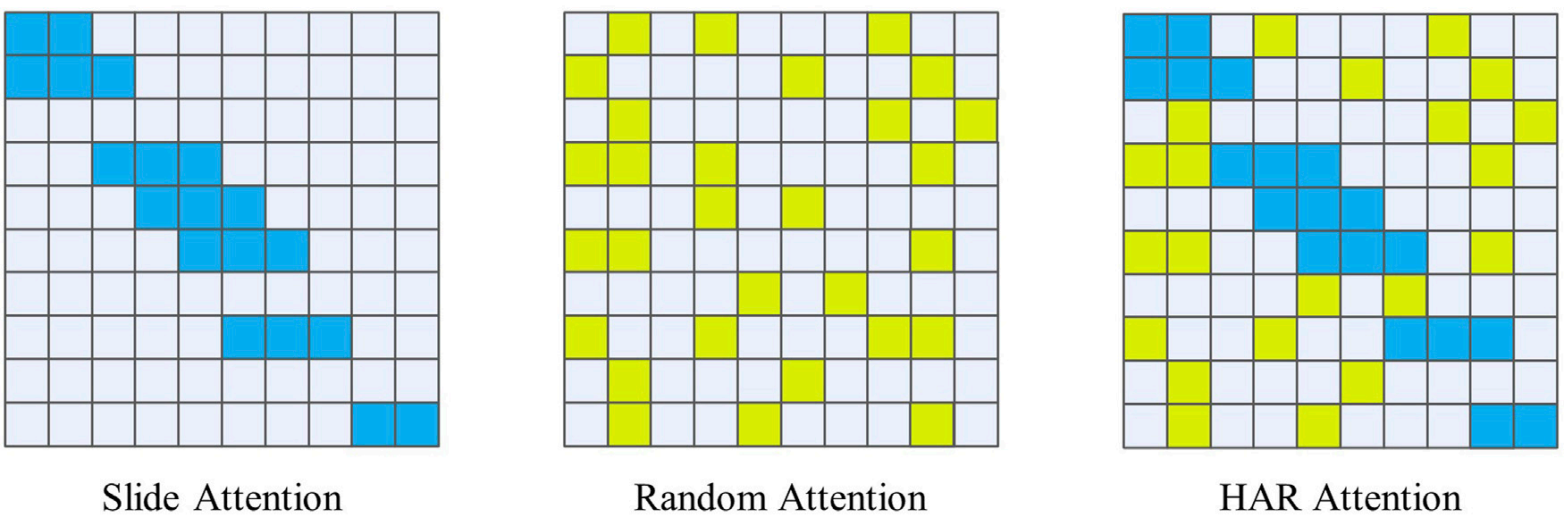
HAR attention.

The BigBird Model (Zaheer et al., 2021) is a new type of natural language processing model that incorporates some unique attention mechanisms. When designing the HAR-Attention, a combination of Slide Attention and Random Attention was used to ensure the model’s ability to extract local and global features while reducing computational complexity, as Figure 3 shown. This paper designs an attention mechanism suitable for the HAR field based on the sparse attention mechanism in BigBird.

The Slide Attention mechanism is a strategy based on sliding windows. When calculating attention weights, it only focuses on a small area around the current position in the input sequence and ignores other parts. In each sliding window of HAR data, places where acceleration changes often contain essential features (Sousa Lima et al., 2019). Therefore, using the Slide-Attention mechanism in areas with large fluctuations can ensure the model’s local perception ability. At the same time, this mechanism helps the model effectively solve the performance degradation problem caused by the global attention mechanism, thus improving the model’s computational efficiency and prediction accuracy.

The Random Attention mechanism is based on random sampling. The model’s computation process randomly selects some positions to calculate attention weights for each time step. Unlike traditional global attention mechanisms, the randomly selected positions can be any position, allowing the model to consider all relevant information without having to compute all the information, significantly reducing computational costs.

By combining the characteristics of these two attention mechanisms, the Transformer model based on HAR Attention has more robust modeling capabilities and can better perform human behavior recognition tasks with higher computational efficiency.

## 4 Experimentation and evaluation

This paper tested the proposed model using three large public datasets commonly used in sensor-based human behavior domains: HARTH (Logacjov et al., 2021), PAMAP2 (Reiss and Stricker, 2012), and UCI-HAR(Bulbul, Cetin, and Dogru, 2018).

### 4.1 Datasets

#### 4.1.1 HARTH dataset

The HARTH dataset (Logacjov et al., 2021) contains records of 22 participants wearing two 3-axis accelerometers for approximately 2 h in a free-living environment. The sensors are attached to the right thigh and lower back. The provided sampling rate is 50 Hz. Activities were annotated frame by frame using video recordings from a chest-mounted camera.

#### 4.1.2 PAMAP2 dataset

The PAMAP2 Physical Activity Monitoring dataset (Reiss and Stricker, 2012) is publicly available on the UCI repository and contains 18 body activities (such as lying down, sitting, walking up/down stairs, *etc.*). The dataset was collected from nine subjects wearing three wireless inertial measurement units (IMUs): one on the dominant arm wrist, one on the chest, and one on the ankle of the dominant side. Each subject was asked to perform 12 activities according to a protocol: standing, sitting, walking up/down stairs, running, and walking. The dataset has 52 dimensions, and acceleration, gyroscope, magnetometer, and heart rate data were recorded from the dominant IMU. Each subject also had a heart rate monitor with a sampling rate of 9 Hz. The IMU has a sampling rate of 100 Hz.

#### 4.1.3 UCI-HAR dataset

The UCI-HAR dataset (Bulbul, Cetin, and Dogru, 2018) was collected with a Samsung Galaxy SII, which was positioned on the subject’s waist. The experiments have been carried out with a group of 30 volunteers within an age bracket of 19–48 years. Each person performed six activities (walking, walking-upstairs, walking-downstairs, sitting, standing and laying). Using the phone’s embedded accelerometer and gyroscope, we captured 3-axial linear acceleration and 3-axial angular velocity at a constant rate of 50 Hz.

### 4.2 Experimental environment and parameter tuning

The training and testing of the proposed algorithm were conducted on a machine with an RTX 3060 GPU and an AMD Ryzen 7 5800H CPU with 32 GB of memory. Our code is based on PyTorch implementation. We used the Adam optimizer with decaying learning rates to speed up the model training process for various datasets. Different initial learning rates were set according to each dataset. As there are highly imbalanced classes in various biological activity datasets, different class weights must be considered based on their sample proportions. Three evaluation metrics were used to comprehensively evaluate the proposed model: accuracy and F1-score (Tang et al., 2022). For different datasets, the sliding window length was set to 100 with a 50% overlap. The initial learning rate was set to 0.0001, and cross-entropy was used as the loss function to train the model with a batch size of 128 for 40 epochs. The percentage of the sparse attention mechanism mask used was 70%.

### 4.3 Experimental results

#### 4.3.1 Results on the HARTH dataset

**FIGURE 4:**
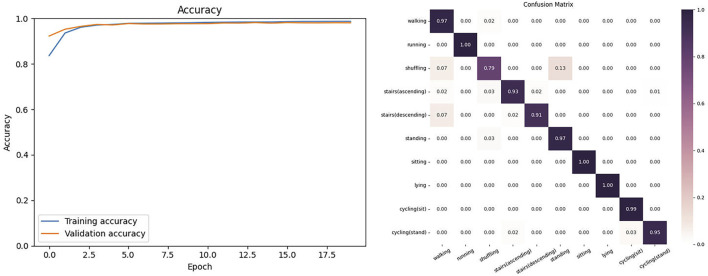
The Accuracy and Confusion matrix on the HARTH.

In this experiment, we selected the six common types of movements in human behavior recognition from the HARTH dataset: walking downstairs, running, sitting, standing, walking, and walking upstairs. We selected the 6-channel acceleration signals collected by two different inertial sensors in the dataset. As shown in Figure 4, the proposed model on the HARTH dataset began to stabilize in accuracy after 20 training epochs and achieved the highest training accuracy after 40 epochs. In this dataset, the model achieved an accuracy of 98.10% and F1-score of 95.29. Figure 4 shows the confusion matrix of the model on the HARTH dataset.

#### 4.3.2 Results on the PAMAP2 dataset

**FIGURE 5:**
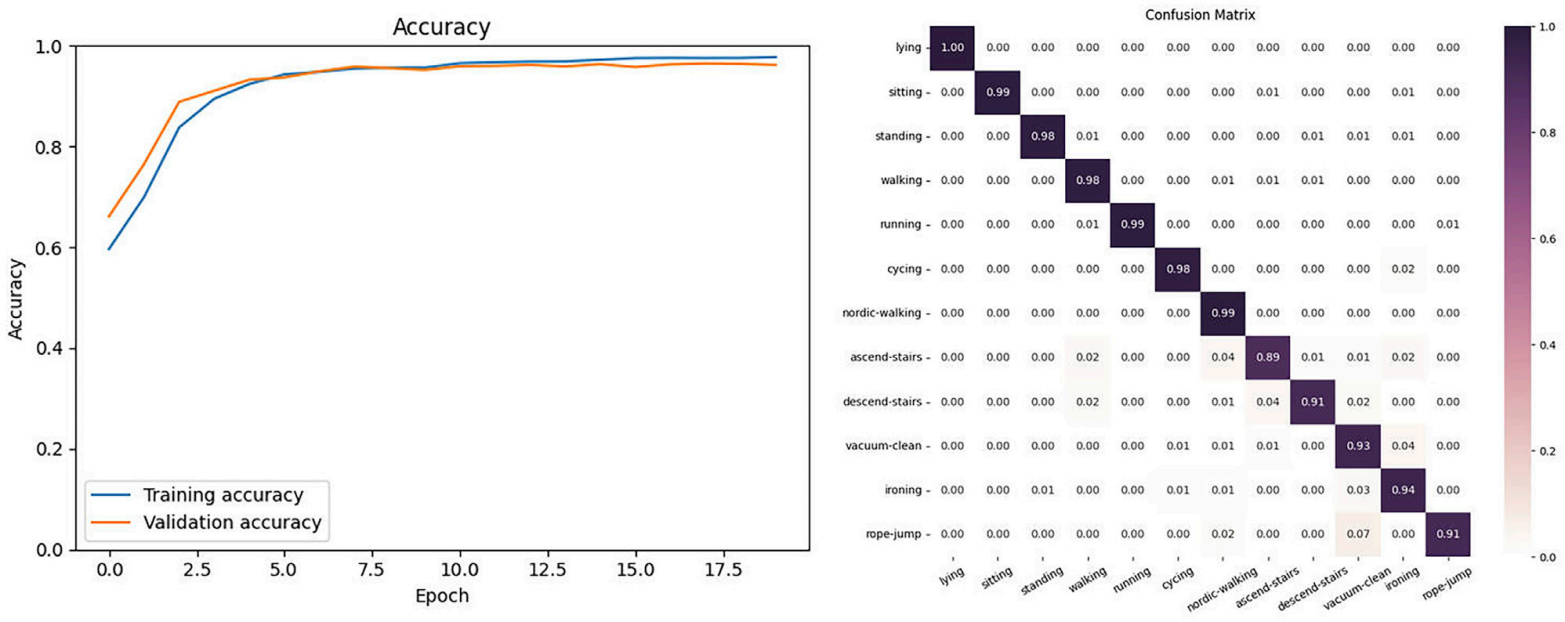
The Accuracy and Confusion matrix on the PAMAP2.

In this experiment, we selected the six common types of movements in human behavior recognition from the PAMAP2 dataset: lying down, sitting, standing, walking, running, cycling, nordic walking, ascending, descending, vacuum cleaning and ironing. We selected the 6-channel acceleration signals collected by two different inertial sensors in the dataset. As shown in Figure 5, the proposed model on the PAMAP2 dataset had tiny fluctuations in its curve after 20 training epochs and achieved the highest training accuracy after 40 epochs. In this dataset, the model achieved an accuracy of 97.21% and F1-score of 96.04. Figure 5 shows the confusion matrix of the model on the PAMAP2 dataset.

#### 4.3.3 Results on the UCI-HAR dataset

**FIGURE 6:**
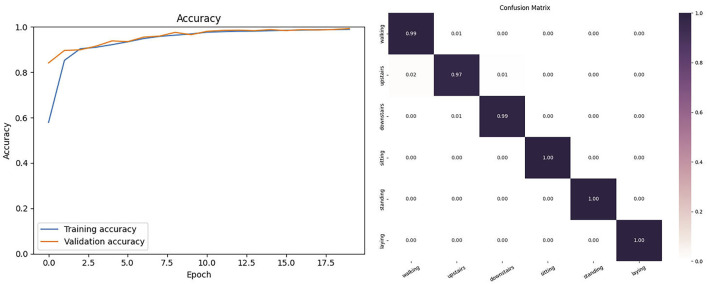
The Accuracy and Confusion matrix on the UCI-HAR.

In this experiment, we selected theactivitys in human behavior recognition from the UCI-HAR dataset: walking, walking-upstairs, walking-downstairs, sitting, standing, andlaying. We selected the 6-channel acceleration signals collected by two different inertial sensors in the dataset. As shown in Figure 6, the proposed model on the UCI-HAR dataset had tiny fluctuations in its curve after 20 training epochs. In this dataset, the model achieved an accuracy of 98.820 and F1-score of 98.22. Figure 6 shows the confusion matrix of the model on the UCI-HAR dataset.

#### 4.3.4 Comparison with related work

In order to evaluate the performance of the proposed method, we compare the proposed method with similar previous research methods. Table 1 summarizes the research methods and corresponding evaluation metrics on the HARTH, PAMAP2, and UCI-HAR datasets in recent related literature, including accuracy (Acc) and F1-score. (Table 1) shows that the proposed model in this paper has the highest accuracy and F1 score on the HARTH dataset. The accuracy of using Multi-Resolution CNN model (MR-CNN) (Hnoohom et al., 2022) is 97.59%, which is 0.51% lower than our method. On the PAMAP2 dataset, the F1 score of our method on the same level asthe most advanced model, and the accuracy using Attention + CNN(Li, Wang, and Liu, 2022) is 97.35%, which is almost the same as the method in this paper, while the accuracy of other methods is lower than 97%.The accuracy of the Multi-branch Neural Network based on Attention-based Convolution (Huan et al., 2022) is 96.01%, which is 1.20% lower than our method. For the UCI-HAR dataset, our method’s accuracy and F1-score are 0.21% and 048.% higher than 1D CNN + Attention (Khan and Ahmad, 2021) and higher than ResNet + Attention (Tang et al., 2022), respectively. In summary, the proposed model in this paper has high accuracy and F1-score, and our method also has fewer parameters and floating-point operations. The Transformer-based human behavior recognition model with an attention mechanism is suitable for complex scenarios, and adding the attention mechanism enhances the effect of adversarial signal interference.

**TABLE 1:** Comparison with rival methods.

HARTH	Method	Acc	F1-score
	LSTM (Logacjov et al., 2021)	97.19	94.03
	CNN-LSTM (Logacjov et al., 2021)	97.45	93.99
	MR-CNN (Hnoohom et al., 2022)	97.59	94.76
	Our network	98.10	95.29
PAMAP2	CNN +GRU (Dua, Singh, and Semwal, 2021)	95.27	95.24
	LSTM+CNN (Xia, Huang, and Wang, 2020)	95.01	95.85
	Attention + CNN (Li, Wang, and Liu, 2022)	97.35	97.03
	Attention + Convolution (Huan et al., 2022)	96.01	95.52
	MhaGNN (Y. Wang et al., 2023)	96.74	96.33
	Our network	97.21	96.04
UCI-HAR	ResNet + Attention (Tang et al., 2022)	98.61	-
	1-D CNN + Attention (Khan and Ahmad, 2021)	98.18	97.72
	CNN + BiLSTM (Nafea et al., 2021)	98.53	-
	Our network	98.82	98.20

### 4.4 Ablation experiment

We conducted the following ablation experiments on the HARTH dataset to test the effectiveness of the channel attention mechanism and sparse self-attention mechanism. We replaced the channel attention mechanism with a continuous 1D CNN. We also compared the use of the sparse self-attention mechanism with its absence.

The experimental results showed that adding the channel attention mechanism enhanced the model’s ability to extract features from data collected by different sensors, enabling the Transformer module to extract more robust contextual features and significantly improving the model’s performance. When using the sparse self-attention mechanism, the model’s accuracy fluctuated slightly. However, the fluctuation was slight, and adding the sparse self-attention mechanism reduced the Flops computational complexity of the model.

### 4.5 Model robustness evaluation

The robustness of the proposed model is evaluated by 5-fold cross-validation method. The detailed experimental scheme is shown below.

**FIGURE 7:**
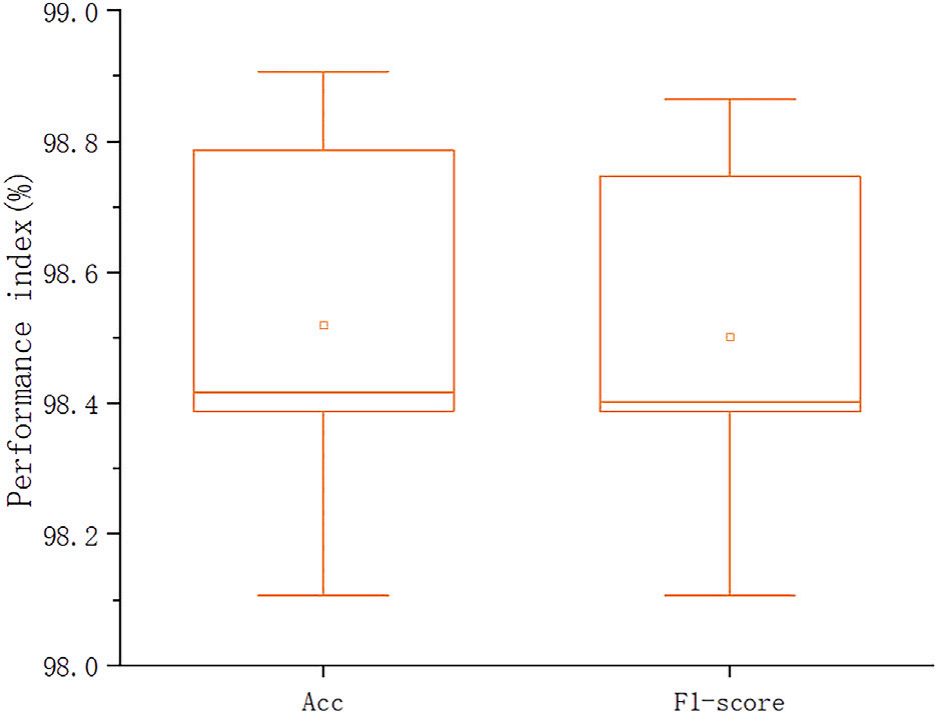
5-fold cross validation results of the model.

5-fold cross-validation method: We divide the entire data set into 5 parts by stratified sampling method. Stratified sampling is to make the data distribution of each part roughly the same as that of the entire data set. Then we select 4 parts of the data as the model training set and the remaining 1 part as the validation set. The results of the 5-fold cross-validation of the hybrid model based on the Channel Attention Mechanism and Sparse Attention Mechanism Transformer are shown in Figure 7, from which it can be seen that each evaluation index is stable within a reasonable range. The accuracy of the model is 98.52% ± 0.29% (average ± standard deviation), the sensitivity is 90.48% ± 1.13%, and the F1-score is 98.5% ± 0.29%. It is further shown that the model has strong robustness and can achieve good performance.

## 5 Conclusion and future work

This study proposes a hybrid model based on the Channel Attention Mechanism and Sparse Attention Mechanism Transformer for human activity recognition tasks on sensor data from wearable devices. The model performed initial feature extraction on multi-dimensional signal data using the channel attention mechanism and 1D CNN and used a sparse Transformer model to extract features from sensor data. In the self-attention module of the Transformer model, we introduced a dual attention mechanism composed of Slide Attention and Random Attention to select the essential features. By analyzing the confusion matrices of the HARTH, PAMAP2, and UCI-HAR datasets, our proposed method can reduce the adverse effects of activity similarity on activity recognition and effectively improve the accuracy of sensor-based HAR. Compared with existing research results, our method outperforms the current state-of-the-art methods.

In the future, we will further investigate the following aspects: 1) consider the transfer learning problem in sensor-based HAR; 2) consider the class imbalance problem in sensor-based HAR.

## Data Availability

Publicly available datasets were analyzed in this study. This data can be found here: https://archive.ics.uci.edu/dataset/231/pamap2+physical+activity+monitoring https://archive.ics.uci.edu/dataset/240/human+activity+recognition+using+smartphones https://archive.ics.uci.edu/dataset/779/harth.
